# The role of elastin on the mechanical properties of the anterior leaflet in porcine tricuspid valves

**DOI:** 10.1371/journal.pone.0267131

**Published:** 2022-05-13

**Authors:** Samuel D. Salinas, Yasmeen M. Farra, Keyvan Amini Khoiy, James Houston, Chung-Hao Lee, Chiara Bellini, Rouzbeh Amini

**Affiliations:** 1 Department of Bioengineering, Northeastern University, Boston, MA, United States of America; 2 Department of Biomedical Engineering, The University of Akron, Akron, OH, United States of America; 3 Department of Psychology, Middle Tennessee State University, Murfreesboro, TN, United States of America; 4 School of Aerospace and Mechanical Engineering, The University of Oklahoma, Norman, OK, United States of America; 5 Department of Mechanical and Industrial Engineering, Northeastern University, Boston, MA, United States of America; Georgia State University, UNITED STATES

## Abstract

Elastin is present in the extracellular matrix (ECM) of connective tissues, and its mechanical properties are well documented. In Marfan syndrome, however, the inability to properly code for the protein fibrillin-1 prematurely leads to the degradation and loss of elastin fiber integrity in the ECM. In this study, the role of elastin in the ECM of the anterior leaflet of the tricuspid valve was investigated by examining the biomechanical behavior of porcine leaflets before and after the application of the enzyme elastase. Five loading protocols were applied to the leaflet specimens in two groups (elastase-treated and control samples). The mechanical response following elastase application yielded a significantly stiffer material in both the radial and circumferential directions. At a physiological level of stress (85 kPa), the elastase group had an average strain of 26.21% and 6.32% in the radial and circumferential directions, respectively, at baseline prior to elastase application. Following elastase treatment, the average strain was 5.28% and 0.97% in the radial and circumferential directions, respectively. No statistically significant change was found in the control group following sham treatment with phosphate-buffered saline (PBS). Two-photon microscopy images confirmed that after the removal of elastin, the collagen fibers displayed a loss of undulation. With a significant reduction in radial compliance, the ability to withstand physiological loads may be compromised. As such, an extracellular matrix that is structurally deficient in elastin may hinder normal tricuspid valve function.

## Introduction

Marfan syndrome (MFS) results from a mutation of the *FBN1* gene that encodes for fibrillin-1, a structural protein that is a component of the microfibrils in the extracellular matrix (ECM) [1]. Along with collagen, elastic fibers are not only abundant but serve as the principal load-bearing components of the ECM. Elastic fibers (or as commonly referred to as elastin fibers) are composed of amorphous elastin cross-linked with microfibrils [2, 3]. Loss of elasticity in the skin or in the blood vessels, for example, are attributed to the degradation and loss of elastin in the body as part of the aging process. While no clear consensus on elastogenesis seems to be established, previous researchers noted that the rate of elastin synthesis can be measured up until puberty [4] or that elastin turnover can be a meager 1% per decade [5]; it remains clear that, with the passage of time, elastin is subject to degradation. Although such loss and degradation of elastin may occur, leading to loss in tissue compliance, elastin remains the most stable molecule in the ECM. In the case of MFS, however, connective tissues throughout the body are adversely affected by the disorder due to a concert of molecular mechanisms that leads to partial loss of the elastic fiber function [6]. From a mechanical vantage point, this is significant in that such microfibrils, of which fibrillin-1 is a structural unit, form an anchoring scaffold that contributes to the structural integrity and mechanical competency of the ECM network. Despite the involvement of other organ systems, the skeletal, cardiovascular and ocular systems are the chief organ systems on which the diagnostic criteria for MFS are focused [3]. It is in these systems that elastin plays a key role in transferring and distributing physiological loads. While not unique to MFS, epidemiological studies have shown that there exist associations between certain ocular and cardiovascular disorders due to the potential role for ECM structural integrity to affect systems in the eyes and heart [7]. For example, there is a higher risk of glaucoma in patients with a history of mitral valve prolapse [8]. The biomechanical role of ECM proteins both in glaucoma and in heart valve disorders is currently a topic of wide interest [9–13]. It is interesting to note that MFS is a risk factor for both valvular prolapse [14] and glaucoma [15], further indicating a potential biomechanical role for ECM elastin in the etiology of these diseases in MFS patients.

Estimates of MFS prevalence range from 1 in 5000 to as high as 1 in 3000 individuals [3, 16] and, although life expectancy has been on the rise (now approaching that of the normal population), serious complications can threaten the lives of those affected. As the heart undergoes more than three billion loading cycles in a lifetime [17], it is important to study the heart closely, given that MFS severely affects this organ. In fact, the highest risk for death posed by MFS involves the cardiovascular system: cardiovascular complications are the primary cause of death in MFS, accounting for up to 95% of fatalities [2, 18–20]. Aortic rupture, for example, remains the most common fatal event in MFS. In the realm of valvular complications, Marfan patients frequently present mitral valve regurgitation [14]. Although less prevalent than mitral regurgitation, tricuspid valve (TV) prolapse and subsequent TV regurgitation also influence morbidity and mortality of Marfan patients, as such complications in the TV often occur concurrently with those in the mitral valve [19, 21].

Presumably because of its location and its relatively lower functional ventricular pressure, the TV was once considered “the forgotten valve” [22] and has often been overlooked [21]. Nevertheless, epidemiological, clinical, and genetic studies have collectively shown that the function of this biomechanically active tissue is closely related to its ECM composition. How ECM elastin fibers contribute to the biomechanical integrity of the TV leaflets has not yet been examined. To bridge this knowledge gap, in our study, we quantified the mechanical properties of porcine TV anterior leaflets in the presence and absence of native ECM elastin by using biaxial tensile testing. While the biomechanical role of elastin has been studied in other tissues (e.g. in carotid arteries [23]), this study is the first one conducted for the TV. To visualize the microstructural architecture of the TV anterior leaflet ECM, we used two-photon excitation microscopy in native and elastase-treated tissues. This study may provide insights that lead to a better understanding the etiology of TV complications when the composition of the ECM proteins are compromised, as is the case in MFS.

## Materials and methods

### Sample preparation

Fresh porcine heart samples, which were obtained from a slaughterhouse (3-D Meats,Dalton, OH) within a one-hour drive of our testing facility, were immediately transported to our laboratory in a cooled phosphate-buffered saline (PBS) solution to maintain tissue integrity. The male-to-female ratio of the pigs providing the tissue samples was unknown; however, all animals were approximately 6 months of age and had a post-processed weight of 180 kg. Although other animal models could be used for this study, the porcine heart is preferred over the hearts of other animals because it is anatomically similar to a human heart. One specific study that involved the TV apparatus also reported that there was no significant difference in the leaflet thicknesses of a porcine valve as compared to a human valve [24].

**Fig 1:**
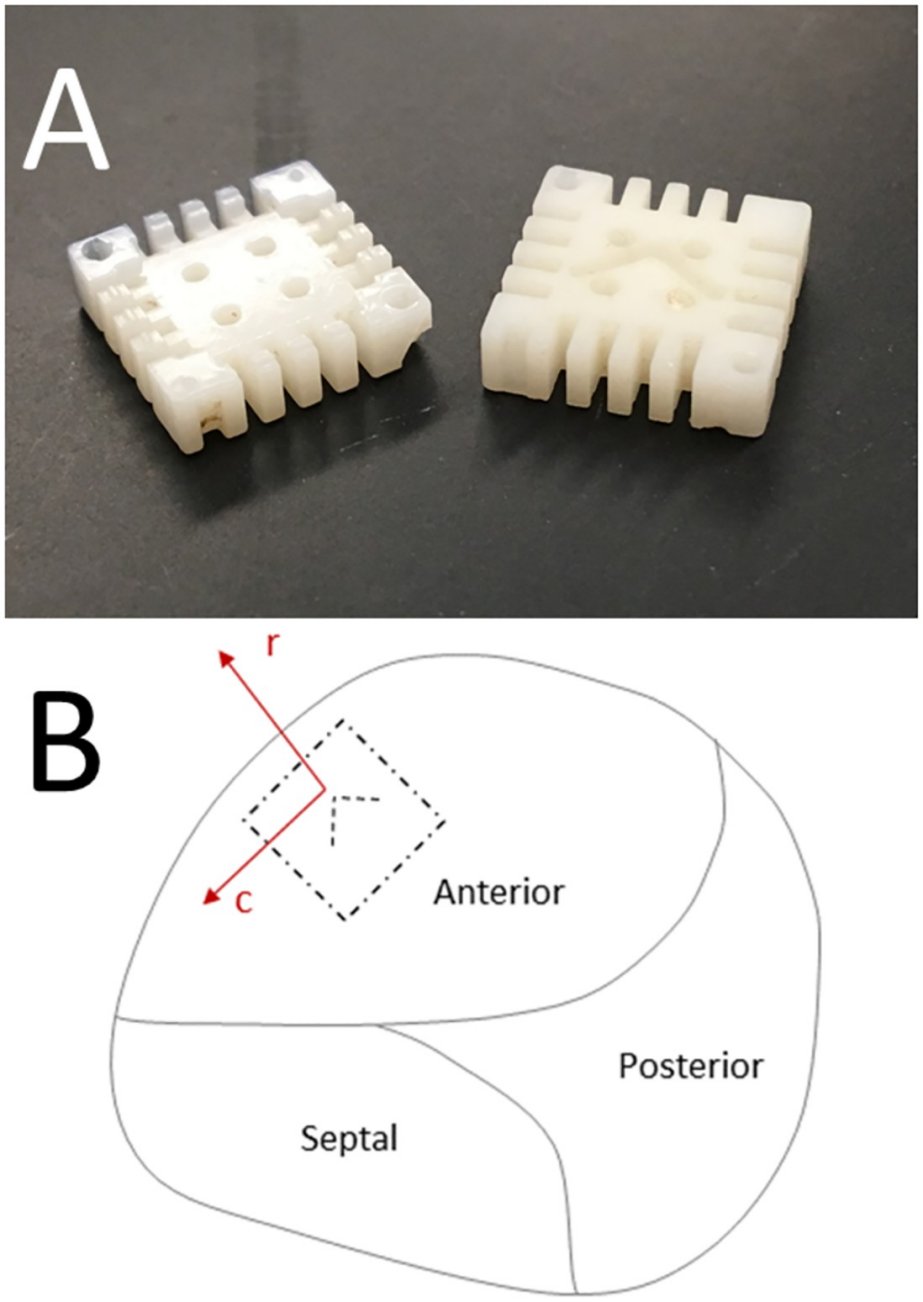
(A) Custom designed 11*mm* × 11*mm* phantom used to trim specimen for use on biaxial testing machine. (B) Position of the tissue phantom, with identified axes used during the biaxial loading tests, in relation to the tricuspid valve anatomy.

Once the porcine hearts arrived at the laboratory, the TV was located and the anterior leaflets were excised. The anterior leaflet was removed with part of the annulus still attached to enable the anatomical orientation of the tissue to be easily identified. We used surgical scissors to further trim the leaflet was further trimmed to dimensions of 11 × 11 mm. The phantom, shown in Fig 1A, has grooves in the top and bottom halves that allow for the tissue to be secured between the two halves while it is carefully trimmed. Close attention was paid to the orientation of the tissue relative to the tissue template. Since we had preserved part of the annulus on our leaflet specimens, we used this anatomical structure to guide the placement of each leaflet on the phantom prior to trimming. The direction tangential to the TV annulus was noted as the circumferential direction and was aligned to one axis of the phantom. The radial direction, which is orthogonal to the circumferential direction, was by default aligned to the remaining axis on the phantom, shown in Fig 1B. Further details of this sample preparation procedure have been previously been described at length. [25–28].

### Biaxial testing

In this study, custom-made biaxial tensile testing equipment (Amini Khoiy and Amini, 2016) was used to stretch specimens in two orthogonal directions under controlled loading, where load cells were used to measure the force applied in each direction independently. More detailed information about this equipment is provided in our previous publication (Amini Khoiy and Amini, 2016).

The highest loading stress applied in this experiment was determined based on the right ventricular pressure of 30 mmHg, the maximum pressure for a normal human heart [29, 30]. The resulting stress experienced by the anterior leaflet was calculated to be approximately 120 kPa using the Laplace approximation as described previously [25];from the same study, we adopted 313 μm as the average thickness of the anterior leaflet. Other than the equibiaxial stress of 120 kPa, four other distinct loading protocols, shown in Table 1, were utilized. As a stress-controlled study, it is important to note that the load applied by the biaxial testing machine was varied for each sample in order to achieve the target stress. This is because, although the effective length of the specimen is 7.6 mm, the cross-sectional area of the leaflet was subject to variation due to the different anterior leaflet thicknesses. As such, the applied load on the specimen would have to be adjusted on a sample-by-sample basis. Throughout the different loading protocols, the tissue was submerged in PBS at a standard room temperature of 21°C. The specimen was subjected to 10 cycles of preconditioning for each loading protocol with a tare load of 0.5 g. The loading–unloading cycle for the protocols was set to 40 seconds. Only data pertaining to the tenth cycle was used for our analysis.

**Table 1:** Radial and circumferential loading protocols.

Protocol	Radial (kPa)	Circumferential (kPa)
1	120	120
2	90	120
3	120	90
4	60	120
5	120	60

### Elastase application

In a process similar to the one used in a previous study [23], purified porcine pancreatic elastase (Worthington Biomedical Corp. Lakewood, NJ, USA; 99% Protein, 11.2 U/mgP) was dissolved in PBS to a concentration of 7.5 U/ml. After applying all loading protocols, the specimen was carefully removed from the biaxial testing machine and submerged in elastase for 20 minutes in order to remove the elastin from the collagen fibers. The attached fiducial markers were not removed during the treatment application so as to preserve the same reference configuration. In other words, we were able to quantify the strain once with respect to the pre-treatment reference configuration and once with respect to the post-treatment reference configuration. In addition, the suture lines were left attached to the specimen during the application time for two reasons. First, by leaving the hooks attached, we avoid having to repeat the initial procedure of attaching the suture lines and risk damaging the tissue when remounting it on the biaxial testing machine. Second, allowing the hooks to remain on the sample offered a visual indicator that prevented a mismatch of axes at the time of tissue remounting.

The elastase-treated specimen was retrieved from the elastase solution after 20 minutes. It was then rinsed and allowed to rest in PBS for another 20 minutes prior to remounting on the biaxial testing machine. The soaking and resting procedure was applied for both the elastase group (*n*_*e*_ = 10) and the control group (*n*_*c*_ = 10); in the latter group, PBS was used in lieu of the elastase solution. The 20-minute elastase application time was consistent with previous research involving carotid artery and atrioventricular valves [23, 31]. We have also shown that no traces of elastin was visually detectable in our examined samples. One could, however, measure the mechanical strain during the gradual digestion of elastin and identify a direct relationship between exposure time to elastase and tissue mechanical responses. Such investigations could be made in future research.

#### Stress and strain calculation

Data analysis was performed by an in-house code in MATLAB (MathWorks, Nantick, MA, USA). The process of calculating two-dimensional surface strains via fiducial markers has been elaborated in previous studies [32, 33]. In our experimental setup, four small glass markers (<1 mm) were placed at the center of our specimen. The deformation matrix from the pixel location of each marker was acquired by the aforementioned technique. Having obtained the deformation gradient tensor, **F**, we used a direct approach to calculate the right Cauchy–Green strain tensor **C**:

$$\begin{array}{c} C=F^{⊤}F \end{array}$$ (1)

One can readily calculate the Green–Lagrangian strain tensor by utilizing previously determined right Cauchy stress tensor **C** and the identity tensor, **I**:

$$\begin{array}{c} E=\frac{1}{2}\left(C-I\right) \end{array}$$ (2)

In our data analysis, we relied on two stress tensors for our calculations and interpretation: the first Piola–Kirchhoff stress **P** and the second Piola–Kirchhoff stress **S**. The normal stress components *P*_*rr*_ and *P*_*cc*_ (in the radial and circumferential directions, respectively) were calculated based on the assumption of planar loads with no shear, as discussed previously [25, 34].

$$\begin{array}{c} P_{rr}=\frac{f_{r}}{A_{o}} \end{array}$$ (3)

$$\begin{array}{c} P_{cc}=\frac{f_{c}}{A_{o}} \end{array}$$ (4)

where *f*_*r*_ and *f*_*c*_ are the forces applied over the undeformed cross-sectional area *A*_*o*_ in the radial and circumferential directions, respectively. The second Piola–Kirchhoff stress, **S**, was then calculated:

$$\begin{array}{c} S=F^{-1}P \end{array}$$ (5)

### Constitutive modeling

With the assumption that the tissue is an incompressible and hyperelastic material, we employed a strain energy function (SEF) to model the response of the anterior leaflet before and after elastin digestion. The second Piola–Kirchhoff stress was then determined by

$$\begin{array}{c} S_{ij}=\frac{\partial W}{\partial E_{ij}} \end{array}$$ (6)

In this study, we modeled the leaflet response solely as a Fung-type model *W* [35]

$$\begin{array}{c} W=\frac{c}{2}(e^{Q}-1) \end{array}$$ (7)

where *Q* is calculated by

$$\begin{array}{c} Q=a_{1}E_{rr}^{2}+a_{2}E_{cc}^{2}+2a_{3}E_{rr}E_{cc} \end{array}$$ (8)

In Eq 8, *c* and *a*_1_–*a*_3_ are material constants; *E*_*rr*_ and *E*_*cc*_ are the normal components of the Green–Lagrangian strain tensor in the radial and circumferential directions, respectively (see Eq 2).

To obtain the parameters of the aforementioned model, the second Piola–Kirchoff stress values were fitted to the stress–strain values obtained from the five experimental biaxial testing protocols in Table 1 by using an in-house MATLAB script that relies on a trust-region-reflective algorithm [36]. The fitted parameters obtained from this process were then compared for the pre- and post-treatment application.

### Microscopy

Images were acquired on an Olympus FV10MP microscope (Olympus Corporation, Tokyo, Japan) coupled with a MaiTai mode-locked tuneable laser (Spectra Physics Inc., Mountain View, CA, USA). Two-photon microscopy was used in this study for two main reasons. Firstly, this imaging modality provides the added depth parameter that allows fresh specimens with a thickness up to 1 mm to be imaged. As our specimens are well within this thickness limit (<0.5 mm), it was possible to obtain images of the leaflet through the thickness. Secondly, the inherent fluorescent properties of collagen and elastin allow for images to be acquired without the need for protein-specific dyes. Both atrioventricular valves have leaflets composed of four layers (atrialis, spongiosa, fibrosa and ventricularis) [37]. The atrialis, on the atrial side of the valve, contains most of the elastin [38]. This layer was visualized by applying an excitation wavelength of 780-nm. The spongiosa and the fibrosa are largely collagen-containing layers in the atrioventricular valves [39]. Collagen fibers were imaged using an 840-nm excitation wavelength, acquired through second-harmonic generation (SHG) luminescence.

In addition to two-photon microscopy, histology was performed to assess the digestion of native elastin following treatment with elastase. Three adjacent samples were isolated from a fresh porcine anterior leaflet. The first sample remained intact without any elastase treatment. The second sample was treated in the elastase solution only for 10 minutes. Finally, consistent with previous published work on carotid arteries [23], the last sample was placed in the elastase solution for 20 minutes. The samples were then fixed in 4% paraformaldehyde overnight and were sent to a company specializing in histology (HORUS Scientific, Worcester, Massachusetts, USA) for processing using Movat Pentachrome stain. The stained sections were then imaged using our bright-field microscope (Leica DM4 B) with a 20x objective.

### Statistical analysis

To differentiate the effectiveness of the treatments on each group, a Wilcoxon signed-rank test was performed. The non-parametric approach was selected for the statistical analysis in lieu of Student’s *t*-test because of the absence of normality among the collected data sets. For example, the exposure to elastase was a source for the non-normal distribution of the radial strain measurements. A threshold of *p* <.05 was selected as a cutoff for determining statistical significance. As higher variation was found for the samples in the elastase-treated group, the Wilcoxon signed-rank test was selected, as it is better suited to identify a treatment effect.

## Results

### Mechanical response

**Fig 2:**
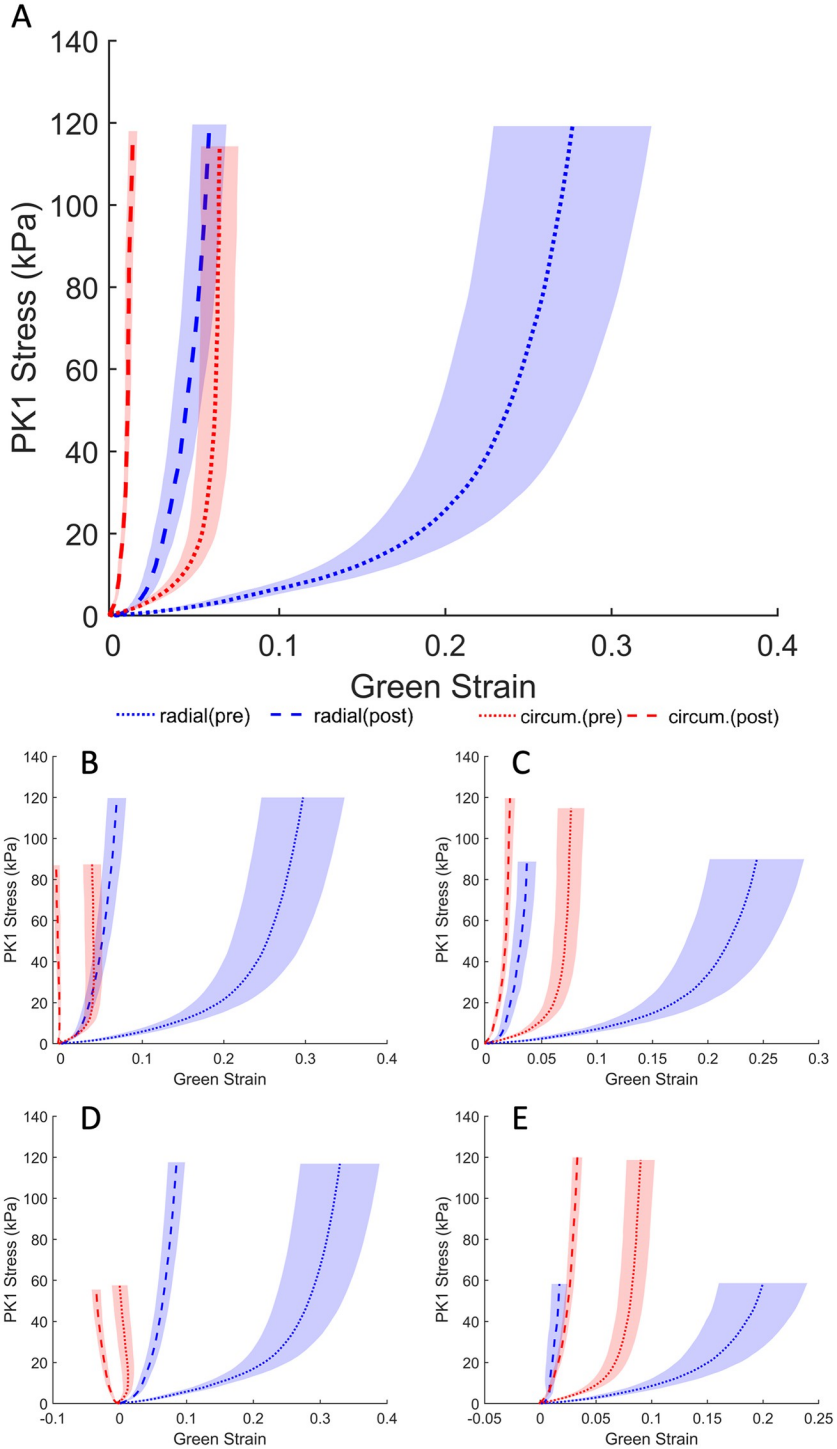
Average first Piola–Kirchhoff response of elastase treatment across the elastase-treated group (*n* = 10). Loading protocols, in kPa (radial—circumferential): (A) 120-120, (B) 120-90, (C) 90-120, (D) 120-60, (E) 60-120. Shaded regions represent standard error.

**Fig 3:**
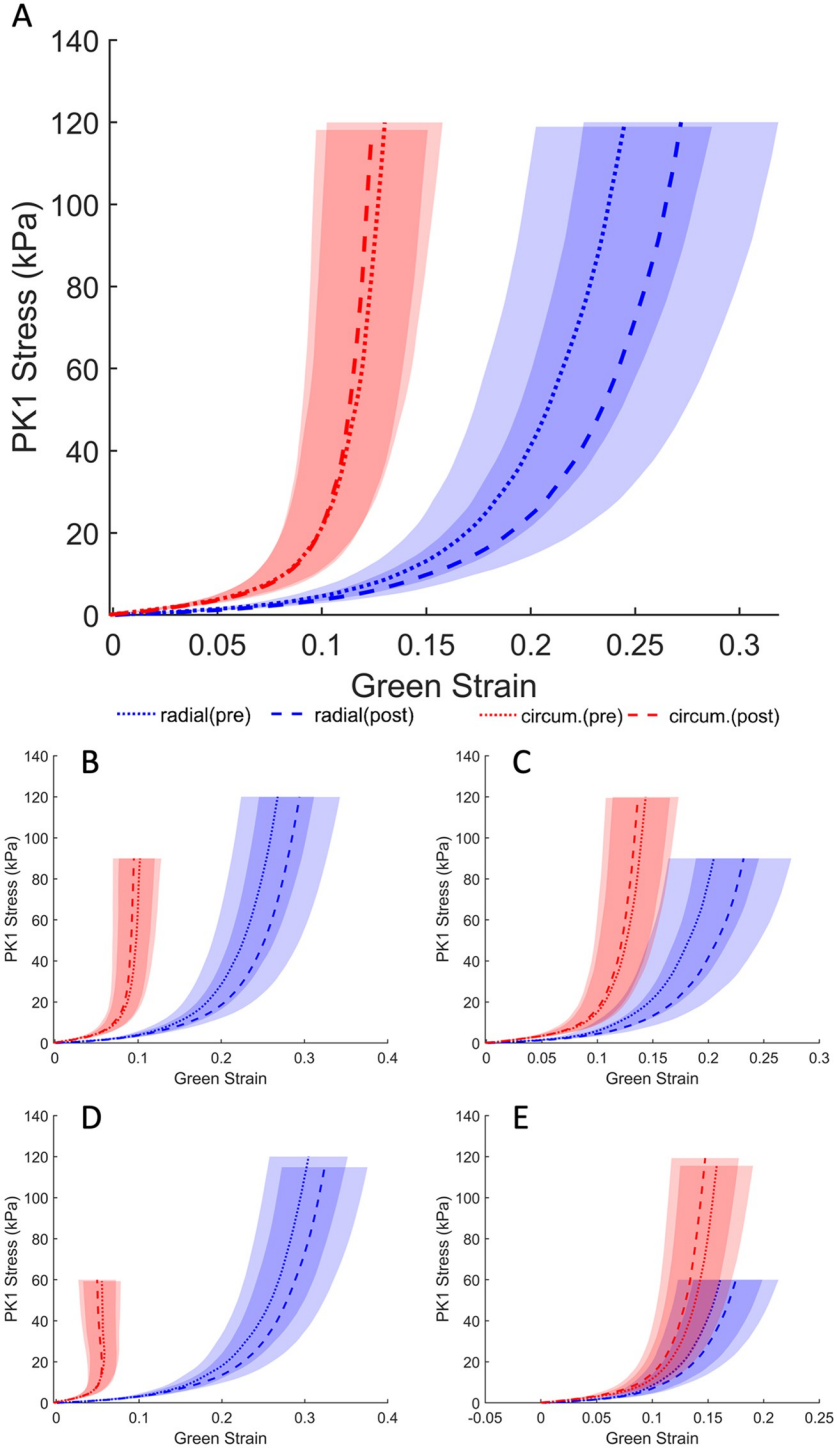
Average first Piola–Kirchhoff response of PBS treatment across control group (*n* = 10). Loading protocols, in kPa (radial—circumferential): (A) 120-120, (B) 120-90, (C) 90–120, (D) 120-60, (E) 60-120. Shaded regions represent the standard error.

**Fig 4:**
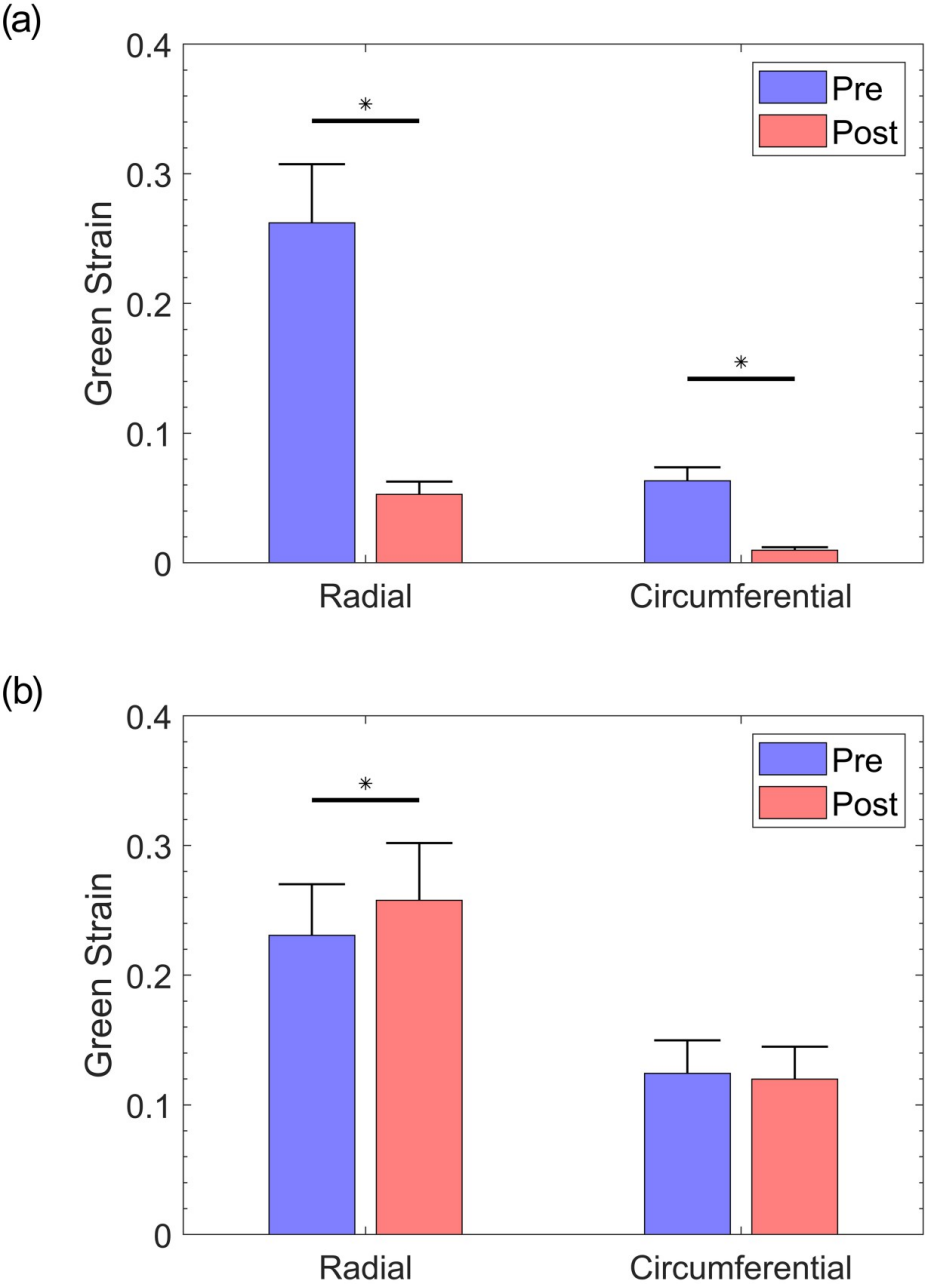
Average strain at 25 mmHg RVP, in the equibiaxial protocol, for a) elastase-treated and b) control groups. Error bars represent the standard error.

The response of the TV leaflet specimens across both groups (elastase-treated and control) showed that the leaflets were more compliant in the radial direction than in the circumferential direction (Figs 2 and 3), which is a result of the tissue’s inherent anisotropy. The anisotropic responses of the untreated tissues were also consistent with previous measurements both in human and porcine valves [25, 40, 41]. Following the enzyme treatment, the elastase-treated group (*n*_*e*_ = 10) demonstrated statistically significant decreases in strain for both the radial and circumferential directions (Fig 2). As expected, there were no significant differences in our control group (*n*_*c*_ = 10) before and after sham treatment. Fig 4 clearly shows a dramatic difference in the elastase group when compared to the control group at a stress level equivalent to 25 mmHg right ventricular pressure (RVP). As discussed in the methods section, with an approximation from the law of Laplace, a normal value of 25 mmHg RVP is equivalent to 85 kPa equibiaxial stress on the anterior leaflet.

Figs 2 and 3 represent an arithmetic average of all sample strains over the same stress for each protocol listed in Table 1. An average response for each protocol could be obtained to represent the general behavior for a non-linear elastic material, as previously described [26]. At the equibiaxial stress level equivalent to 25 mmHg RVP for the elastatse-treated group prior to elastase treatment, the radial and circumferential strain were 26.21% ± 4.53% (mean ± standard error) and 6.32% ± 1.05%, respectively. These values are an order of magnitude higher than the post-treatment strains of 5.28% ± 0.97% and 0.97% ± 0.23% at the same stress level for the radial and circumferential directions, respectively. When compared to the pre-elastase strains, the post-elastase strain values were statistically different for the radial (*p* <.001) and circumferential directions (*p* <.05). Fig 4 illustrates the differences for both groups and their respective treatments. The reduced strain on the specimen not only applies at high stress values; it can also be observed at stress levels below the normal RVP. At a lower stress level (i.e., 1 kPa), the average pre-elastase strains in the radial and circumferential directions were 2.65% ± 0.51% and 0.62% ± 0.22%, respectively. Following treatment application, strains of 0.76% ± 0.17% and −0.11% ± 0.05% for the radial and circumferential directions, respectively, were recorded. Once again, statistical significance was found for strains in both the radial (*p* = .0017) and circumferential directions (*p* = .0036).

In the control group, for which PBS was used in lieu of elastase, strains before treatment were 23.07% ± 3.95 and 12.42% ± 2.56 in the radial and circumferential directions, respectively. Again, after PBS treatment, average strains of 25.77% ± 4.43 and 11.98% ± 2.51, were observed for the radial and circumferential directions, respectively. Statistical testing revealed that at a physiological pressure of 85 kPa, the circumferential strains were not significantly different (*p* = .695), while the radial strains were found to be significantly different (*p* = .027). At the lower stress level (i.e., 1 kPa), the radial and circumferential strains were 3.59% ± 0.55% and 1.33% ± 0.35%, respectively, prior to PBS treatment. Post-PBS treatment results were 4.28% ± 0.54% and 1.66% ± 0.36% for the radial and circumferential directions, respectively. Once again, no statistical significance was found for either the radial (*p* = .105) or the circumferential direction (*p* = .275).

### Constitutive modeling

**Fig 5:**
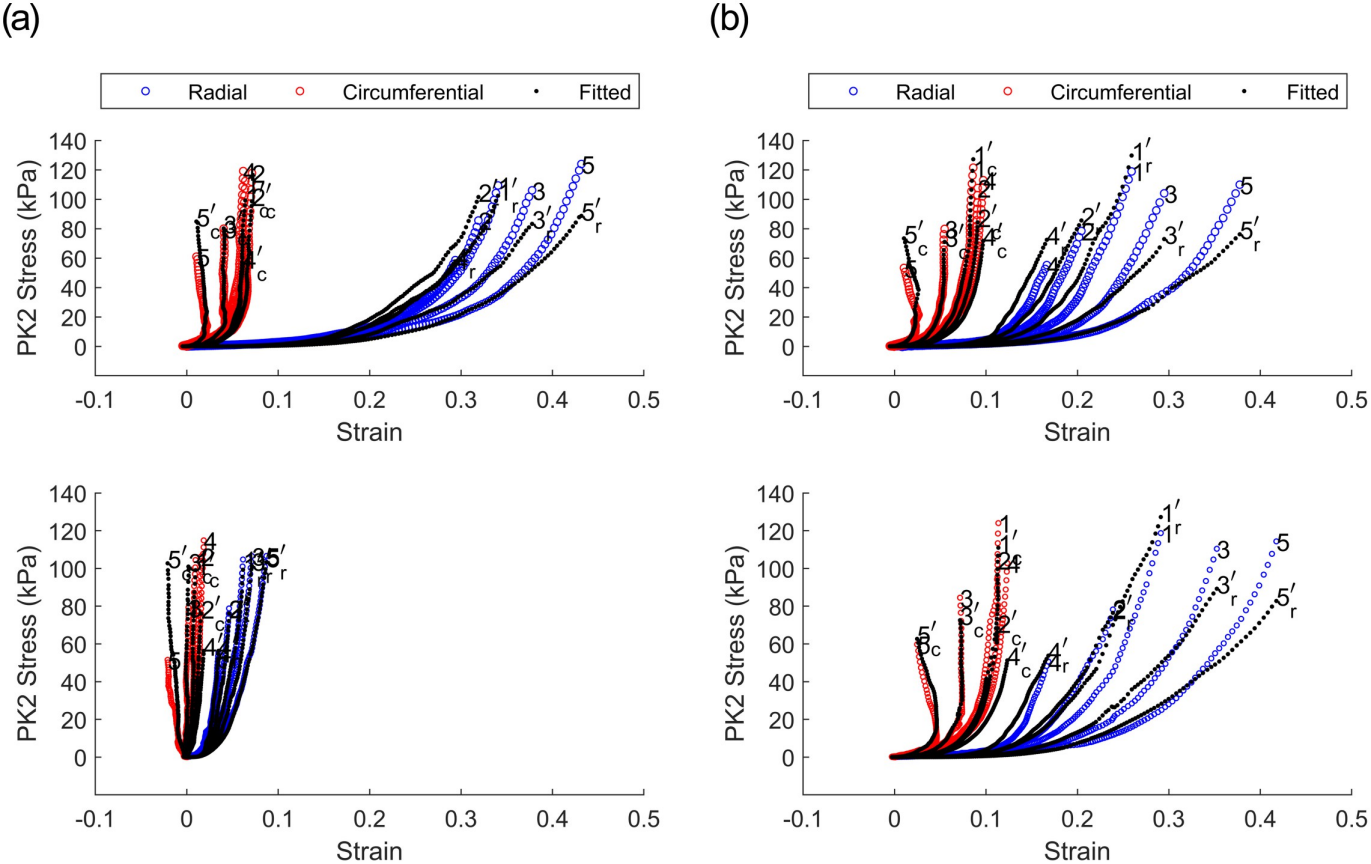
Data fitting to a typical heart in both groups utilizing the Fung-type model for a) elastase-treated and b) control group. Fitting for data prior to treatment (top) compared to after receiving treatment application (bottom). Single number represents the protocol while the number with the added prime represents the optimized fit result.

**Fig 6:**
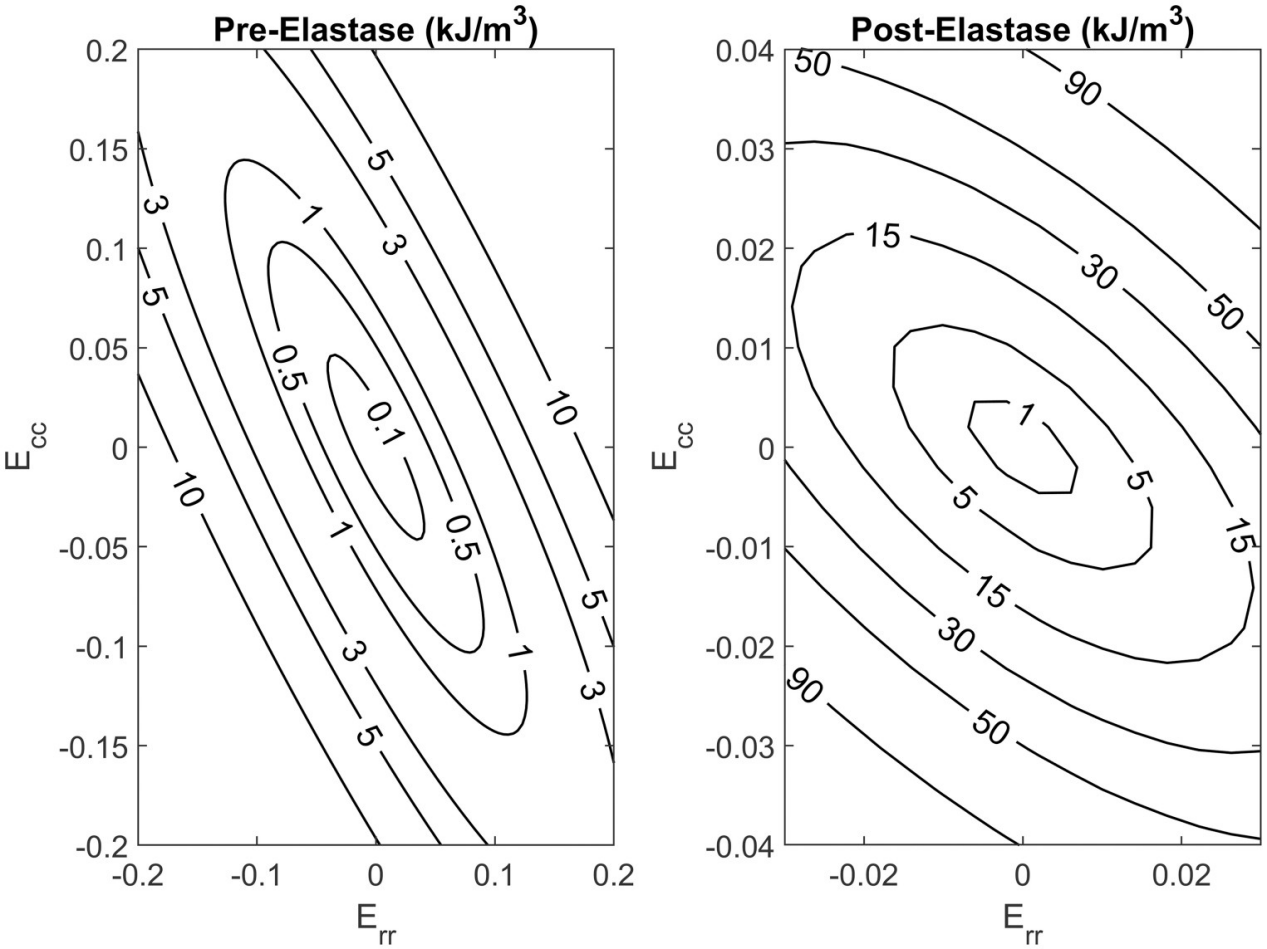
Strain energy density contours, generated before and after elastin digestion, for a typical heart in the elastase treated group.

**Fig 7:**
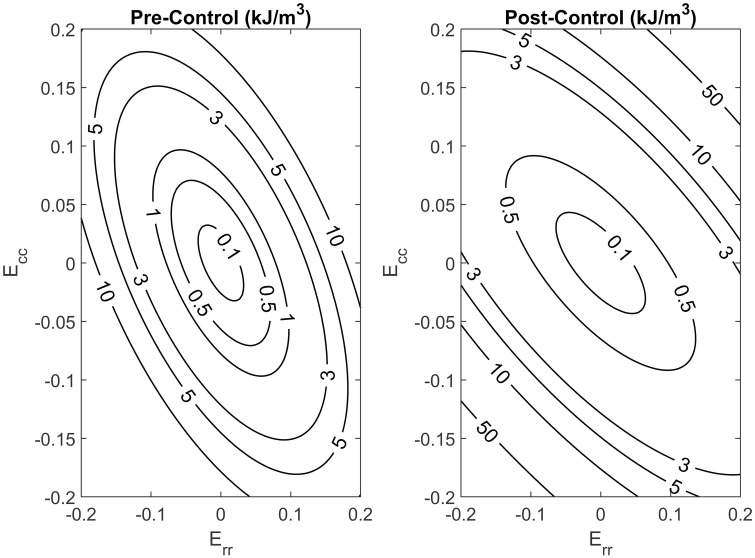
Strain energy density contours, generated before and after PBS treatment, for a typical heart in the control group.

The stress and strain calculations in this study were fitted to the acquired data using a Fung-type model (Fig 5). Fitting parameters for the Fung-type strain energy model from Eqs 7 and 8 are listed in Tables 2 and 3 for the pre-elastin scenario and the post-elastin degradation scenario, respectively. The findings for the pre- and post-sham treatment fit parameters in the control group are listed in Tables 4 and 5, respectively. There is a clear difference in the fitting parameters for the two groups, as expected, and most of the *c* parameters in the post-elastase scenario have higher values. The parameter *c* in the control group seemed to maintain a constant value in the same vicinity regardless of PBS application, as expected. To further examine the uniqueness of the fitting parameters, we tested for convexity in the specimen through a given strain range. This approach is also appropriate for generating reliable simulations for finite element analysis [42]. Strain energy contours were then generated for each specimen in both treatment groups. With the exception of one heart in each treatment group, the graphs demonstrated that convexity was present for a typical heart in this study, as seen in Figs 6 and 7.

**Table 2:** Pre-elastase fitting parameters for Fung-type strain energy function.

Heart	*c* (kPa)	*a*_1_	*a*_2_	*a*_3_	*R* ^2^
1	4.53	9.03	135.82	8.62	0.8927
2	4.93	22.48	60.55	11.65	0.8978
3	47.16	2.11	17.80	2.33	0.8312
4	5.28	6.85	47.80	6.44	0.8757
5	23.10	28.52	289.79	30.32	0.9177
6	21.62	28.28	0.0282	18.90	0.8255
7	20.31	16.96	13.60	17.07	0.9238
8	11.83	14.08	21.72	6.49	0.9246
9	7.84	27.94	45.44	7.68	0.9351
10	4.77	7.35	118.39	7.41	0.8797

**Table 3:** Post-elastase fitting parameters for Fung-type SEF.

Heart	*c* (kPa)	*a*_1_	*a*_2_	*a*_3_	*R* ^2^
1	30373	2.91	13.65	2.90	0.8539
2	25475	4.23	11.00	4.04	0.8448
3	16716	2.54	5.86	2.29	0.8432
4	7397.4	2.85	4.84	2.74	0.8163
5	12077	7.80	12.91	8.03	0.8788
6	15510	11.84	14.95	9.82	0.7178
7	12924	8.39	4.67	4.09	0.8688
8	18063	7.03	11.84	4.10	0.9739
9	2798.1	20.52	17.58	11.29	0.9785
10	2097.3	24.88	36.29	18.95	0.9436

**Table 4:** Pre-sham treatment (control group) fitting parameters for Fung-type SEF.

Heart	*c* (kPa)	*a*_1_	*a*_2_	*a*_3_	*R* ^2^
1	2.66	19.23	35.01	10.49	0.8765
2	6.60	43.72	43.20	25.85	0.8996
3	4.87	36.21	19.64	15.02	0.9268
4	7.40	4.50	89.33	3.15	0.9121
5	13.44	14.70	9.19	13.63	0.9212
6	0.4541	30.28	54.44	9.08	0.94
7	5.14	10.26	131.31	9.86	0.9219
8	0.308	19.99	22.12	13.86	0.9450
9	6.67	8.26	128.06	8.15	0.8798
10	38.44	2.93	205.04	2.82	0.9230

**Table 5:** Post-sham treatment (control group) fitting parameters for Fung-type SEF.

Heart	*c* (kPa)	*a*_1_	*a*_2_	*a*_3_	*R* ^2^
1	1.25	18.87	85.54	15.8	0.8904
2	3.24	63.34	28.92	30.16	0.8964
3	38.85	18.90	16.04	3.98	0.9384
4	13.63	3.43	71.28	2.69	0.8561
5	5.74	19.32	11.81	16.06	0.9156
6	4.91	12.05	20.41	8.52	0.8550
7	6.72	7.83	75.12	5.72	0.9055
8	0.788	21.16	19.44	2.57	0.8886
9	2.32	12.30	280.06	12.05	0.9026
10	10.02	3.90	285.39	3.68	0.8974

### Microscopy

**Fig 8:**
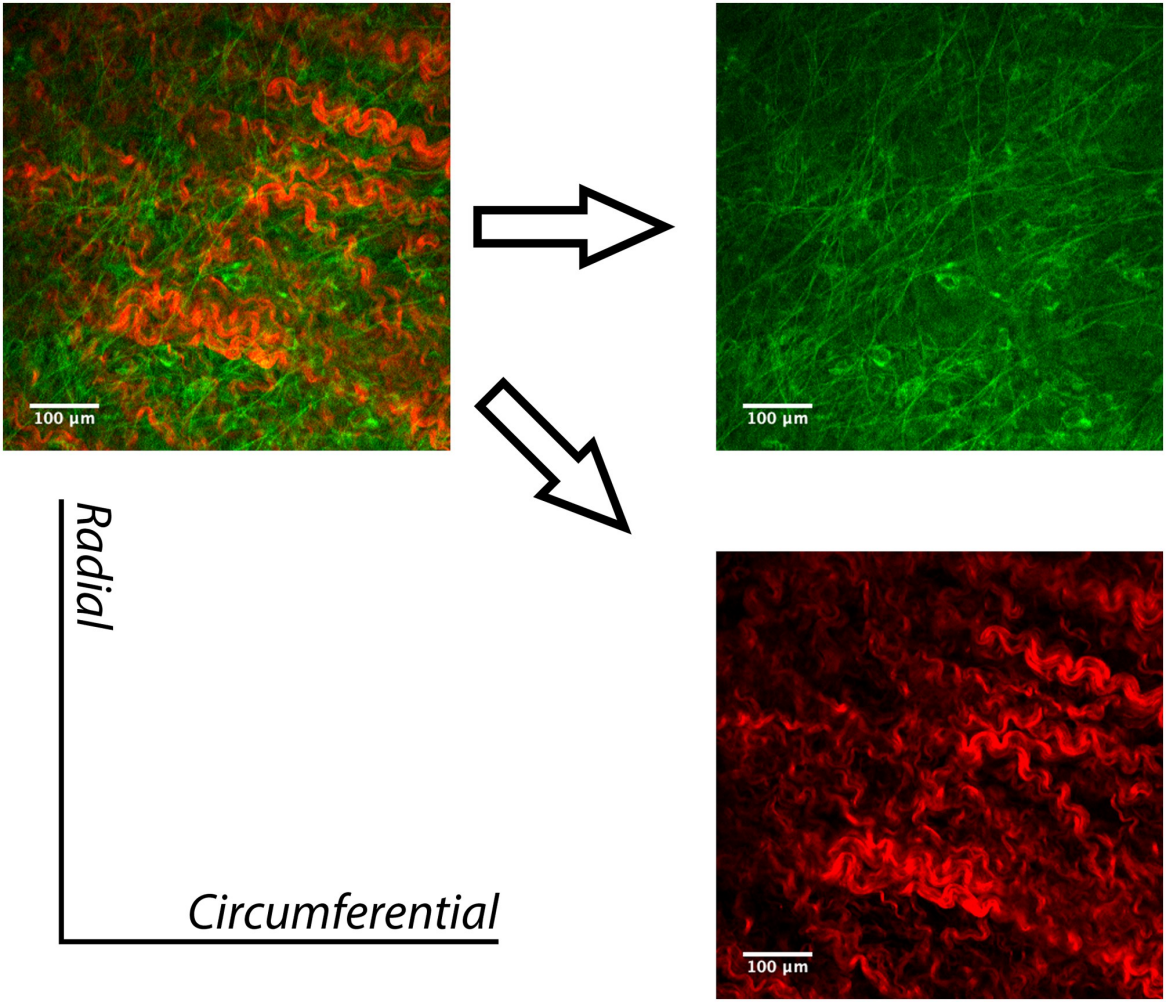
Two-photon imaging (Z-projection) of a typical TV anterior leaflet in the control group. The separate collagen (red) and elastin (green) channels allow for better visualization of collagen and elastin fiber networks.

**Fig 9:**
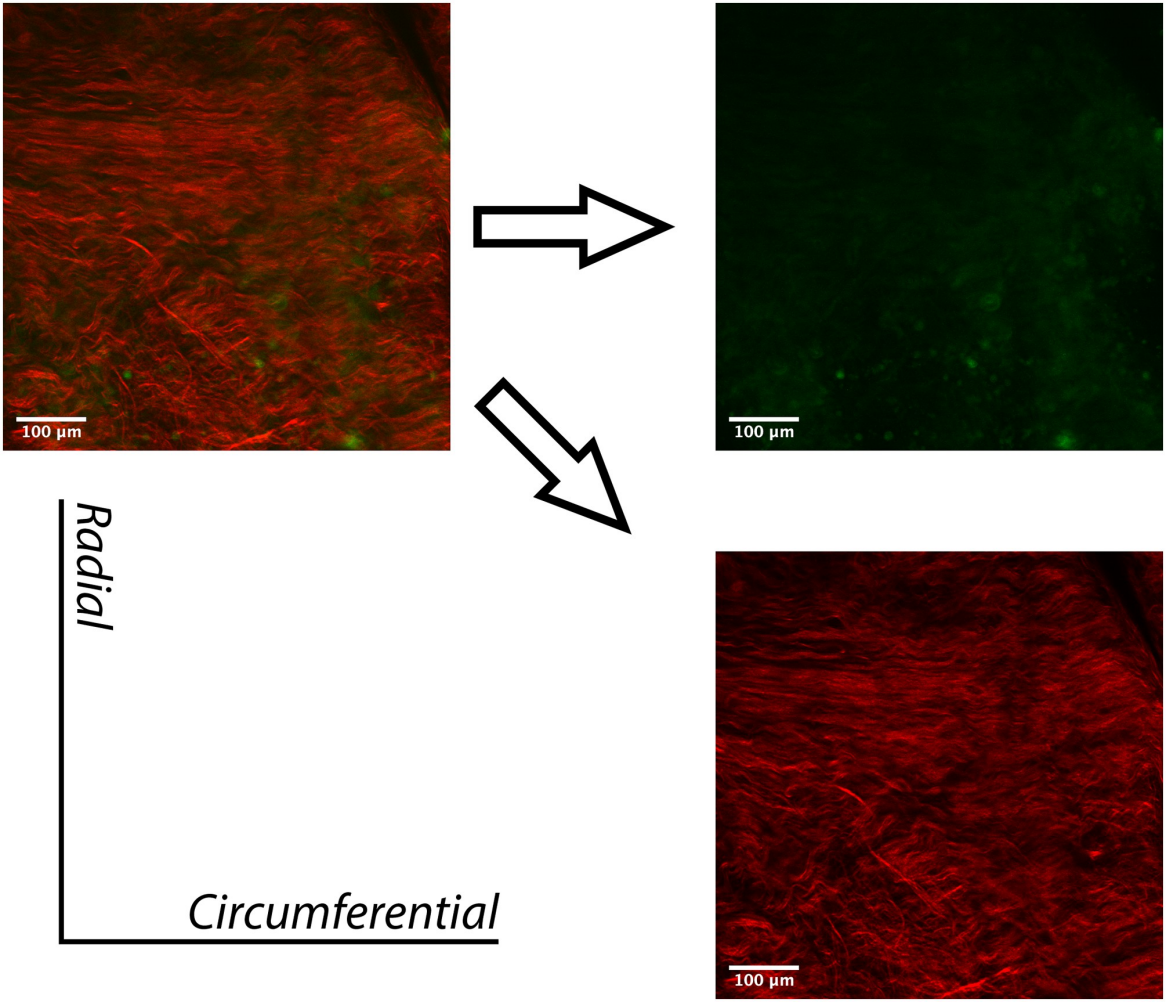
Two-photon imaging (Z-projection) of a typical TV anterior leaflet in the elastase group following elastase application. Elastin fibers are no longer present, therefore only the altered collagen (red) fiber configuration is visible.

A two-photon image of a typical TV anterior leaflet is presented in Fig 8. In this image, collagen is displayed in red with an 840-nm excitation wavelength, as acquired through second-harmonic generation (SHG) luminescence. The orientation of the elastin fibers, from the top to the bottom of the image, demonstrate a preferred alignment towards the radial direction in this specific sample. Similarly, the collagen fiber network has an alignment that is approximately orthogonal to the elastin network. In the case where elastin has been digested, as shown in Fig 9, the alignment of the collagen fiber network seems to have maintained its initial alignment, albeit with a loss of collagen undulation. To ensure that no trace of elastin remained following the 20 minutes of enzyme application, the laser power was increased. However, only noise due to interference was visible, and no structure or organization was present and/or recognizable.

**Fig 10:**
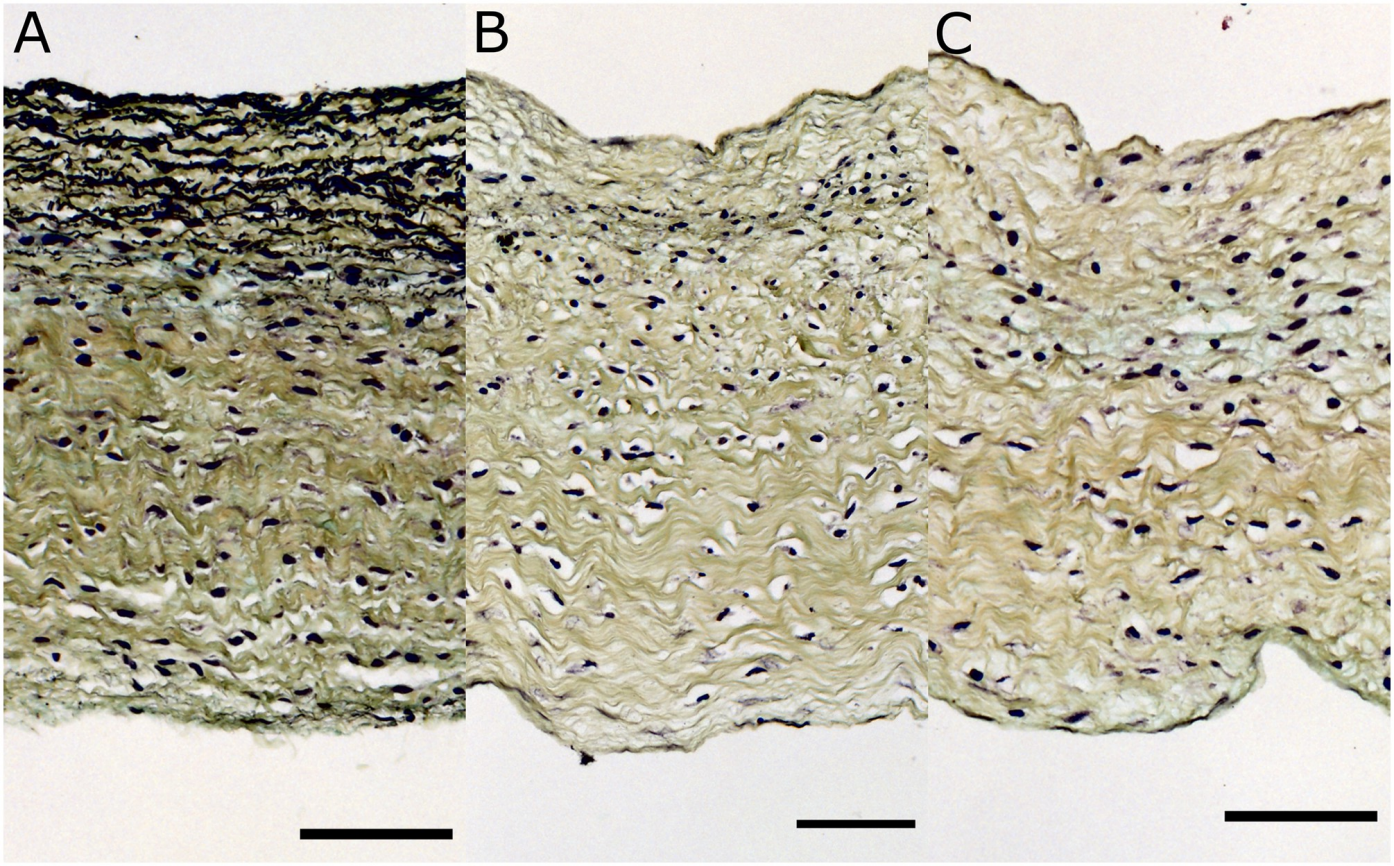
Cross-sectional view of a representative TV anterior leaflet along the thickness: (A) Control, (B) 10-minute elastase digestion, and (C) 20-minute digestion. After Movat Pentachrome staining, the elastin fibers are stained black while the collagen fibers are yellow. The nuclei of the valve interstitial cells (unrelated to this study) are also black. Scale bar represents 75 *μm*.

Further, histology was performed to complement the two-photon microscopy imaging. Three bright-field images of an anterior leaflet stained with Movat Pentachrome are shown in Fig 10A–10C. The elastin fibers are stained black while the collagen fibers are yellow. The nuclei of the valve interstitial cells (unrelated to this study) are also black. As shown in Fig 10A, elastin fibers are visible and are more abundant in the atrialis layer of the native leaflet (towards the top surface in the image). It is visually clear that most of the elastin fibers are fragmented after 10 minutes of treatment with elastase, as almost no residual fiber structure is identifiable in the corresponding 10-minute digestion image (Fig 10B). Following 20 minutes of elastase treatment, no elastin fibers can be visually identified in the image Fig 10C.

## Discussion

The purpose of this study was to analyze the effects of elastin loss on the mechanical response of the TV anterior leaflet in the presence of physiologic stresses. Although previous studies have investigated the effects of elastin on various tissues (e.g., carotid arteries, aorta, and mitral valve [23, 43–46]), this study is the first of its kind to characterize the effects of elastin removal on the TV, specifically the anterior leaflet. In testing the mechanical response prior to and after exposure to elastase, a noticeable stiffening of the tissue was observed once the elastin was digested. Our statistical approach to finding significance between both groups used the Wilcoxon signed-rank test. When analyzing response variables across all samples (the average is shown in Fig 4), it was found that not all datasets were normally distributed. As such, the Wilcoxon signed-rank test was deemed an appropriate and pragmatic method for analyzing all data in this study.

The pre-elastase behavior of leaflets in the elastase group displays a higher compliance in the radial direction as compared to the circumferential direction, suggesting that fiber alignment in each direction is different. It is worth noting that the elastic fiber configuration is not the sole contributor to the mechanical response, but rather is coupled to that of the collagen [47]. The contribution of elastin is further influenced by the overall content present in the tissue [4]. In atrioventricular valves (i.e., the mitral valve and the TV), the elastin network is well established in the atrialis layer, which is located on the inflow side of the valve [17, 39]. The higher stiffness in the circumferential direction is likely due to the higher number of collagen fibers aligned in that direction, as displayed in the images in Figs 8 and 9 [38, 48–50].

The significant mechanical response following elastase treatment can be attributed to the loss of undulation in the collagen fibers, a finding that is consistent with those of a previous study that were verified with multiphoton microscopy [23]. When elastin in native ECM is digested, the collagen fibers are released from their crimped state. This relaxation of collagen fibers, which can be attributed to the modification of a network that was previously untouched, highlights the importance of the elastin–collagen interaction [47]. Previous research focusing on the effect of elastin on the ECM of aortic walls, aortic valves, and carotid walls has found corroborating evidence regarding implications for connective tissues within the cardiovascular system [23, 43–45]. In particular, these studies have shown that elastin allows for a higher compliance under physiological stresses, and the lack of elastin leads to an increased risk in mortality as cardiovascular complications begin to develop. The onset of morbidity demonstrates that collagen, despite being the primary and abundant load-bearing ECM component, is not able to effectively withstand *in-vivo* stresses without the synergy of the additional ECM components.

**Fig 11:**
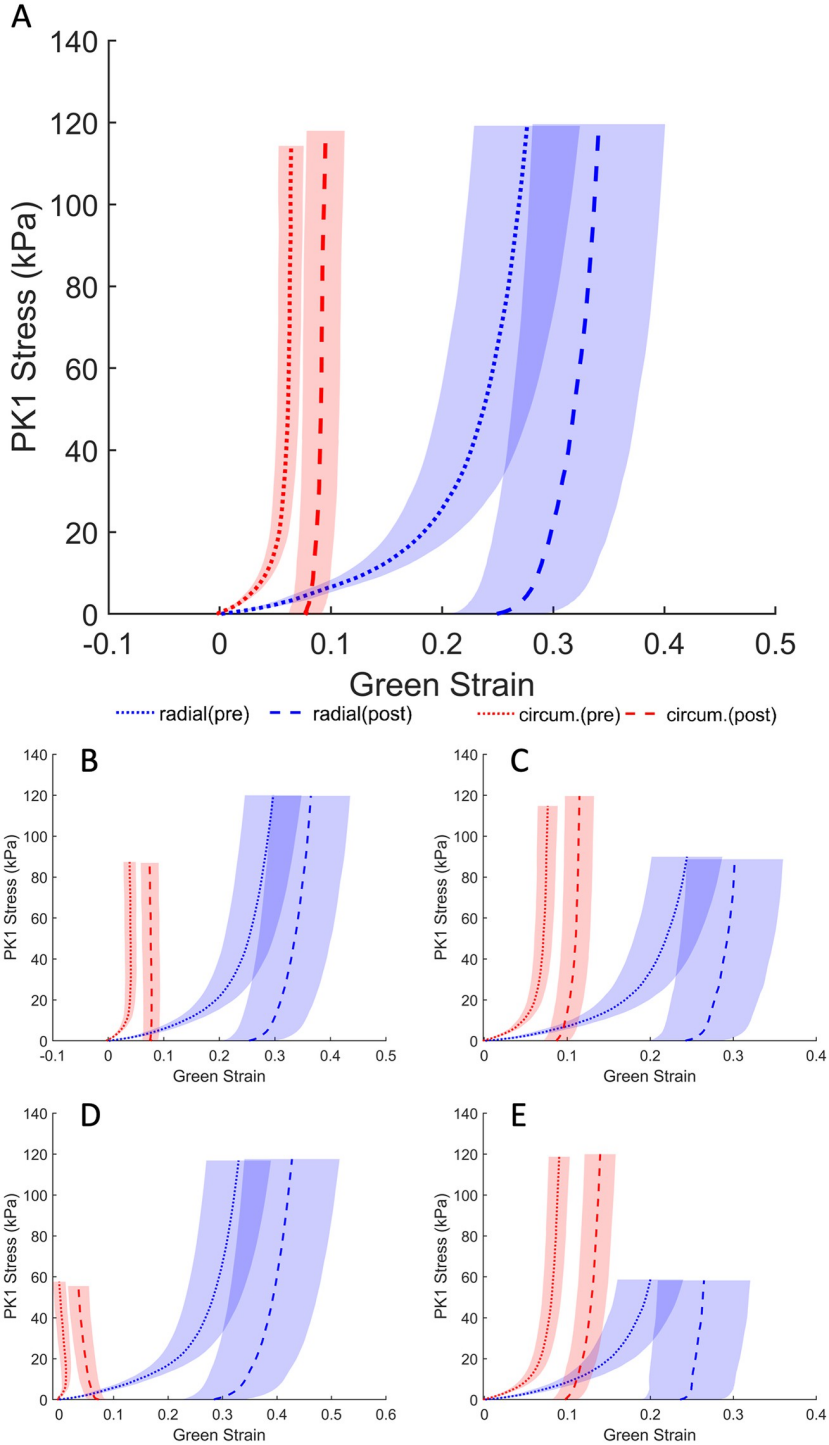
Average first Piola–Kirchoff response of post-elastase samples that reference fiducial marker positions prior to the application of elastase. Loading protocols, in kPa: (A) 120-120, (B) 120-90, (C) 90, 120, (D) 120-60, (E) 60-120. Error bars represent the standard error.

**Fig 12:**
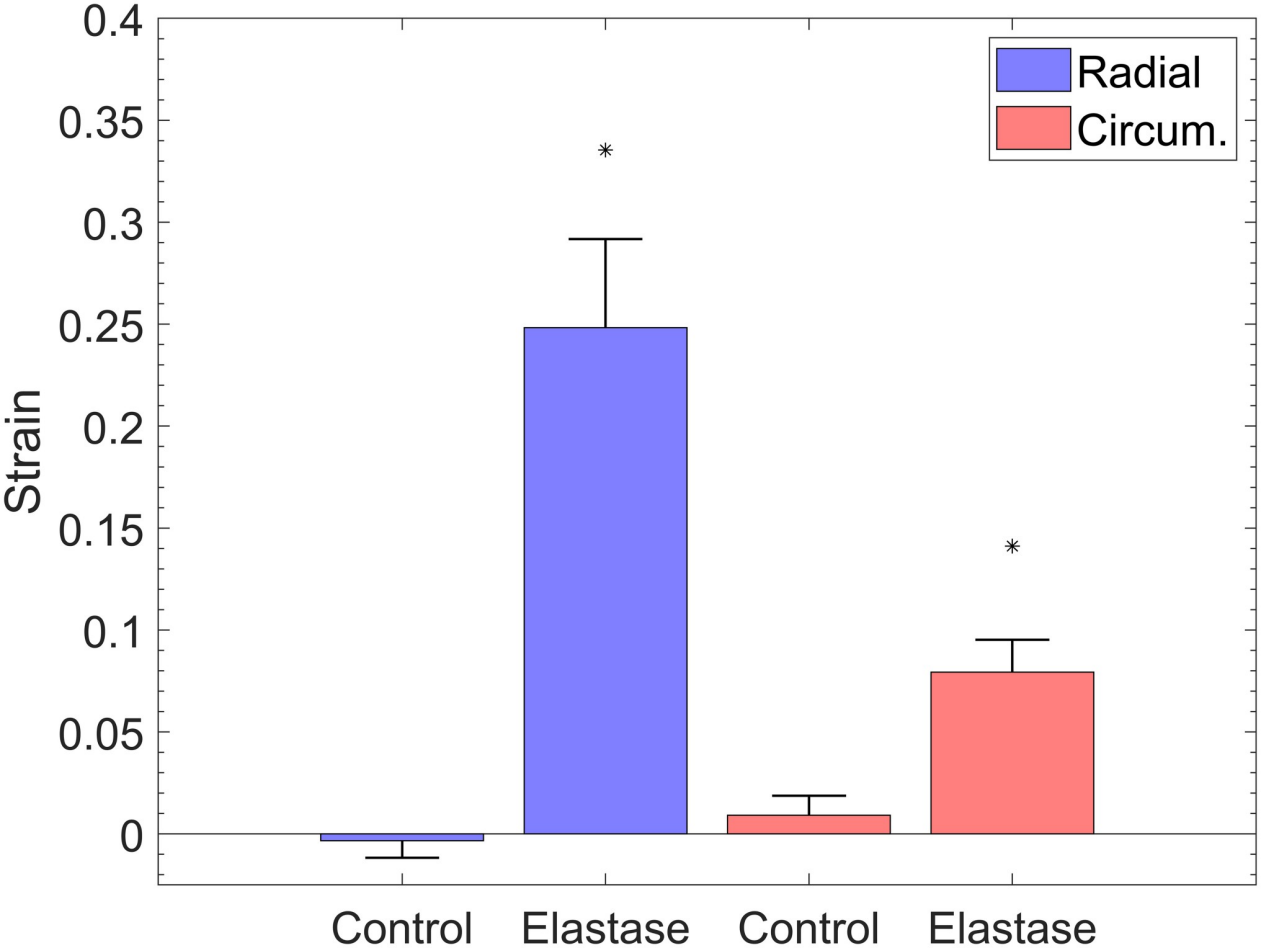
Average strain increase following elastase digestion with reference to pre-elastase marker position at 85 kPa stress level. Asterisk denotes significance (*p* <.05). Error bars represent the standard error.

The bulk of elastin contribution takes place at low pressures, allowing collagen to act as the primary load-bearing fiber at higher pressures [51]. As noted above, the loss of elastin and the accompanying elastin–collagen interaction results in an ECM with a relaxed collagen structure. This modified ECM architecture is hence manifested as a stiffer tissue response (Fig 2). To this end, Fig 3B, for example, displays an increased stiffness in the circumferential direction relative to the Fig 3D. While this response may appear counterintuitive, we remind the reader that the cycle time for any given protocol is set to 30 seconds. As such, regardless of the target load (e.g., 60 or 90) the strain rate will vary and influence the mechanical response. A better visual aid for the role of elastin can be appreciated from Fig 11. While the strain shown in Fig 2 was calculated with respect to the post-elastase tare load, the strain in Fig 11 was calculated using the position of the fiducial markers at the pre-elastase testing tare load. The displayed shift to the right (for the post-elastase scenario) takes into account the strain released as a result of the loss of collagen undulation. The corresponding representation of this elastase-induced shift for both treatment groups (i.e., elastase and control groups) is provided in Fig 12. This figure presents the average strain values following treatment application at the initial tare load prior to the application of any tension. Since the strains at this level are all calculated with the marker positions in the pre-treatment scenario as the undeformed referential frame, both the radial and circumferential directions showed a significant elongation due to changes in the ECM resulting from the addition of the elastase.

Deviations from the native configuration result in a compromised ECM structure in the TV that is, to date, not well understood. As the TV closes during systole, the leaflets coapt. The loss in the undulation of collagen could signify that the load is being transferred to a structure that is mechanically restricted in its ability to accommodate such a physiological demand. There is evidence suggesting that the disruption of the native valve ECM induces signaling pathways that lead to degeneration in the ECM, giving rise to valve disease [52]. As mentioned earlier, mitral valve regurgitation concomitant with TV regurgitation has been been previously reported [19–21]. Diseased TV leaflets in MFS patients were documented to be long enough to protrude into the right atrium [20]. Gu et al. have also documented that for all MFS patients with TV involvement, 100% of the cases involved all three leaflets [21]. Medical management of MFS regurgitation is relevant not only when considering the repair or replacement of a mitral (or tricuspid) valve, but also when considering the higher risk of mortality associated with TV surgical procedures [53, 54].

Our pilot study of two-photon microscopy and bright-field imaging showed that the 20-minute time period for treatment with elastase adopted from previous studies [23] was sufficient to remove elastin structures from the leaflets. In addition, visual examination of the collagen structure in both imaging modalities showed no deterioration of the collagen architecture. Scheriefl et al. have previously shown that elastase treatment could affect collagen structure [55]. However, they used elastase with much higher enzymatic activity (100 U/ml) and for longer digestion time (3 hours) compared to our study (7.5 U/ml for 20 minutes). Although elastin was the main focus of this study, the microscopy images presented also display the inherent morphology of collagen before and after elastin digestion. In future studies, metrics on collagen undulation may be quantified and analyzed with examination of additional samples. Previously researchers have quantified collagen fiber undulation in leporine arteries, and in ovine optic nerves using various imaging modalities and successfully integrated them into models [56, 57].

Our study was not without limitations. One of the limitations relates to the response demonstrated in both the radial and circumferential directions. Although careful attention was given to the way the specimen was trimmed from the tissue sample, there can be no guarantee that the tissue phantom was accurately aligned with the radial direction. Moreover, the varying morphology of the leaflet makes it challenging to maintain the alignment of the specimen. As the tissue is clasped between the two phantom pieces for trimming, variations in thickness from the underlying chordae tendinae may increase the likelihood of the sample shearing and/or rotating from its target alignment. The quantification of the shear strains, however, revealed that such limitations would not be a major concern. In particular, the mean values of the absolute maximum shear strain were 0.038 and 0.0044 for the intact and elastase-treated samples, respectively. Such values were much smaller than the corresponding mean values of the maximum normal strains (0.27 and 0.058 for the intact and elastase-treated samples, respectively).

In the realm of constitutive models of soft tissues, different approaches have been employed to predict the mechanical responses. In this work, we used a phenomenological model similar to other published works [26, 35, 58–60]. However, structurally-based models, in which, the distribution of the fibrous network are included, could also be used for prediction of the soft tissues responses [61]. In some structural models, the undulation of the collagen fibers could also be accounted for in a form of a recruitment function [62, 63]. Such more sophisticated models could be used in future investigations. In order to identify a singe set of parameters for a cohort of tested tissues, one might use the arithmetic average of the fitted parameters presented in Tables 2–5. We, however, advise against such approaches. As we have shown in our previous publication, it is best to first average the experimental data with the same level of stress and then fit those average responses to the model [50].

As discovered in our control group from Fig 4, the unexpected significance in the radial direction within the control groups could be indicative of the potential variance present in our treatment groups. Likewise, such variance may also account for the difference in strain in the circumferential direction between both elastase-treated and control groups. Although one may expect the radial and circumferential strains in these two groups to be comparable, the control group displays a higher strain than the elastase-treated group. The source of existing variance could be attributed to the size of the heart itself, since our TV samples were obtained from a pool of male and female pigs that were approximately 6 months of age.

In addition, each experiment required an average of four hours to complete. Although the hearts were collected and tested on the same day, some error might have been generated due to the deterioration of tissue over the course of the experiment. We also observed that the strain at high stress values in the radial direction differed significantly in the control group post-treatment. Though not expected, this significant difference could be a result of tissue creep following the first set of loading protocols (as observed previously [27, 28]) or because of the degradation of the tissue. Lastly, the responses of the tissue following elastin digestion were based on an ECM structure comprised of collagen that has experienced a sudden loss of undulation. As such, a direct comparison to MFS, which is a congenital defect, is difficult to establish. The collagen fibers in MFS patients have been able to adapt and are able to meet physiological demands—albeit with limited collagen undulation. Despite a current paucity of data on the tricuspid valve mechanical properties of MFS patients, as these become more evident in future studies it will be salient to examine how they differ from tissues that have been treated with elastase. In addition, comparison among tissues with different level of elastin digestion and their similar counterparts in MFS tissues, could provide insights about more clinically relevant responses. In addition, comparison among tissues with different level of elastin digestion and their similar counterparts in MFS tissues, could provide insights about more clinically relevant responses.

## Conclusion

In this study, we have demonstrated how the removal of native elastin affects the mechanical response of the TV anterior leaflet. Our study also provides insights into how damage in elastin, as seen in MFS patients, can play a role in TV competency. It should be noted that while the information and key findings from this study pertain to a porcine model, a correlation can be made to MFS in human counterparts. To the best of our knowledge, this is the first study on the role of elastin in MFS as it pertains to the anterior leaflet of the TV. Our results showed a significant loss in compliance in both the radial and circumferential directions upon digestion of the native elastin. The control group, as expected, did not display a marked change in response since the collagen undulation had been preserved. This study not only highlights the importance of elastin for proper TV biomechanics but also underscores the increased need for further TV biomechanical investigation as it pertains to the medical management of MFS.
